# Na^+^-Dependent High-Affinity Nitrate, Phosphate and Amino Acids Transport in Leaf Cells of the Seagrass *Posidonia oceanica* (L.) Delile

**DOI:** 10.3390/ijms19061570

**Published:** 2018-05-24

**Authors:** Lourdes Rubio, Delia García-Pérez, María Jesús García-Sánchez, José A. Fernández

**Affiliations:** Department of Biología Vegetal, Campus Teatinos, Universidad de Málaga, 29071 Málaga, Spain; lrubio@uma.es (L.R.); delia.garcia.perez@gmail.com (D.G.-P.); mjgs@uma.es (M.J.G.-S.)

**Keywords:** *Posidonia*, high-affinity transport, NO_3_^−^ uptake, Pi uptake, Na^+^-dependent transport systems, Cytosolic Na^+^, amino acid transport

## Abstract

*Posidonia oceanica* (L.) Delile is a seagrass, the only group of vascular plants to colonize the marine environment. Seawater is an extreme yet stable environment characterized by high salinity, alkaline pH and low availability of essential nutrients, such as nitrate and phosphate. Classical depletion experiments, membrane potential and cytosolic sodium measurements were used to characterize the high-affinity NO_3_^−^, Pi and amino acids uptake mechanisms in this species. Net uptake rates of both NO_3_^−^ and Pi were reduced by more than 70% in the absence of Na^+^. Micromolar concentrations of NO_3_^−^ depolarized mesophyll leaf cells plasma membrane. Depolarizations showed saturation kinetics (*Km* = 8.7 ± 1 μM NO_3_^−^), which were not observed in the absence of Na^+^. NO_3_^−^ induced depolarizations at increasing Na^+^ also showed saturation kinetics (*Km* = 7.2 ± 2 mM Na^+^). Cytosolic Na^+^ measured in *P. oceanica* leaf cells (17 ± 2 mM Na^+^) increased by 0.4 ± 0.2 mM Na^+^ upon the addition of 100 μM NO_3_^−^. Na^+^-dependence was also observed for high-affinity l-ala and l-cys uptake and high-affinity Pi transport. All together, these results strongly suggest that NO_3_^−^, amino acids and Pi uptake in *P. oceanica* leaf cells are mediated by high-affinity Na^+^-dependent transport systems. This mechanism seems to be a key step in the process of adaptation of seagrasses to the marine environment.

## 1. Introduction

Nitrogen and phosphorus are essential nutrients for plants, whose availability may limit biomass maintenance and growth. Seawater is a high salinity and alkaline medium with low concentrations of N and P, often below 5 μM in the Mediterranean Sea [1]. The seagrass *Posidonia oceanica*, is a Mediterranean endemism limited by N and P [2], but able to grow under nutrient concentrations low enough (less than 5 μM of dissolved inorganic nitrogen and 1 μM Pi) to limit the proliferation of other species, as *Cymodocea nodosa* [2,3]. This raises the question of how seagrasses are able to extract nutrients at the low concentrations present in seawater to maintain primary production. To take up nutrients at low external concentrations, plants have to: (i) evolve transport systems with the capability to bind the free ion species with a very low *Km* and (ii) a powerful system to energize the movement of the ions across the plasma membranes to compensate the high, outwardly directed, ion motive force. Seagrasses studied so far use the Na^+^ electrochemical gradient to drive the high-affinity uptake of NO_3_^−^ and P_i_ in both roots and leaves [4,5].

In terrestrial vascular plants, where the H^+^-ATPase energizes the plasma membrane [6], NO_3_^−^ transport depends on the cellular energy supply and is coupled to the proton electrochemical gradient [7]. NO_3_^−^ uptake by single cells is associated with depolarization of the plasma membrane, i.e., an increase in the positive charge inside the cell [8]. To account for both the membrane depolarization and the coupling with the proton gradient, it has been proposed that NO_3_^−^ uptake is mediated by a 2H^+^/NO_3_^−^, and therefore electrophoretic, symport mechanism [9,10].

On the other hand, terrestrial plants have evolved a range of physiological and morphological responses which may enhance Pi acquisition under limiting conditions (through symbiotic strategies, root architectural changes, extrusion of organic acids and acid phosphatases by roots, reviewed by [11,12,13]. As in the case of NO_3_^−^, transport of inorganic phosphate requires energy and is also driven by the proton electrochemical gradient generated by the plasma membrane H^+^-ATPase [14,15].

In contrast to terrestrial plants, seagrasses can take up mineral nutrients through roots and also through leaves. However, there are several evidences which indicate that leaf tissues have higher affinity for nutrient uptake and can substantially contribute to total nutrient acquisition ([16] and references therein). As a vascular plant, *Z. marina* plasma membrane is energized by a H^+^-ATPase [17]; nevertheless, unlike terrestrial vascular plants, high-affinity NO_3_^−^ and Pi transport mechanisms are fueled not by H^+^ but Na^+^ in both, root and mesophyll leaf cells [4,5]. Na^+^-coupled transport systems had been previously described in marine organisms, as in the case of the Na^+^-dependent HCO_3_^−^ uptake in marine cyanobacteria [18,19,20] or the Na^+^-dependent NO_3_^−^, glucose and amino acids uptake systems in marine diatoms [21,22]. In addition, phosphate transport has been reported to be stimulated by Na^+^ in several green algae [23]. However, no functional evidences, apart from the case of *Z. marina*, of Na^+^-dependent high-affinity NO_3_^−^ and Pi uptake systems are found in vascular plant literature.

The use of Na^+^ as driving ion for nutrient uptake depends on the maintenance of an inwardly directed electrochemical potential for Na^+^. *Z. marina* maintains homeostatic concentrations of Na^+^ around 10 mM [5] and does it in two ways, first by restricting the plasma membrane permeability to Na^+^ [17] and second by the operation of an Na^+^/H^+^ exchanger that takes Na^+^ out from the cytosol [24]. Thus, living in the presence of 500 mM Na^+^, a membrane potential below −160 mV guarantees an inwardly directed Na^+^ motive force almost 3-fold higher than that for H^+^ at the seawater pH [4,5]. Consequently, a Na^+^-coupled transport system could be an important achievement for seagrasses to colonize the marine environment. Recently, we have reported in *P. oceanica*, the operation of a fusicoccin sensitive H^+^-ATPase that provides a highly negative membrane potential (−174 mV) and the direct HCO_3_^−^ uptake in a symport with H^+^ [25]. Interestingly, *P. oceanica* mesophyll leaf cells exhibit a cytosolic Na^+^ concentration similar to that reported for *Z. marina* (16 ± 1 mM; [25]) sustaining a high inwardly directed Na^+^ driving force that could be exploited to fuel high-affinity nutrient uptake.

We have used the information obtained by classical electrophysiology, ion selective intracellular microelectrodes for Na^+^ and classical depletion experiments to gain insight into the mechanisms and kinetics of the high-affinity transport systems for NO_3_^−^, Pi and amino acids (alanine and cysteine) in the mesophyll leaf cells of the marine angiosperm *P. oceanica*. This species shows high-affinity uptake mechanisms for the nutrient investigated, consistent with the ecological nutrient concentrations described for the Mediterranean Sea; the mechanisms for NO_3_^−^, Pi and amino acids uptake in *P. oceanica*, as previously described for *Z. marina*, are dependent on the presence of Na^+^ (not H^+^) in the medium, which separates this species from the H^+^-based nutrient uptake model that holds for terrestrial vascular plants. Thus, nutrient transport coupled to the electrochemical gradient for Na^+^ could be the general mechanism for high-affinity nutrient uptake energization in seagrasses.

## 2. Results

### 2.1. Effect of Na^+^ on NO_3_^−^ and Pi Net Uptake in P. oceanica Leaves

As aquatic plants, seagrasses can take up nutrients not only through roots but also through the leaves (reviewed by [26]). In the case of *Z. marina*, Na^+^ dependence of high-affinity NO_3_^−^ and Pi uptake have been previously demonstrated [4,5]. In order to analyze if this Na^+^ dependence also occurs in other seagrasses, NO_3_^−^ and Pi depletion assays were performed in *P. oceanica* leaves incubated in artificial seawater with or without Na^+^. In artificial seawater (ASW) containing 500 mM NaCl, N-starved *P. oceanica* leaves completely depleted external NO_3_^−^ (100 μM) after 22 h of incubation, while in the absence of Na^+^, the external concentration of NO_3_^−^ remained around 80 μM at the end of incubation. NO_3_^−^ uptake rate, calculated as the slope of the depletion curves, was 4-fold higher in the presence than in the absence of Na^+^ (Table 1). Similar results were found in the case Pi uptake assays. In ASW containing 500 mM NaCl, P-starved leaves depleted external Pi (10 μM) at 4 -fold higher uptake rate than in the absence of Na^+^ (Table 1). These results strongly suggest the Na^+^-dependence for the NO_3_^−^ and Pi transport in *P. oceanica* leaf cells, to further investigate those transport mechanisms electrophysiological and intracellular ion selective measurements were performed. 

### 2.2. Na^+^-Dependent High-Affinity NO_3_^−^ and Amino Acid Uptake in Single Mesophyll Leaf Cells

Ion transport across membranes leave an electrical trace such as membrane depolarizations when the net charge that enters the cells is positive. Such membrane depolarizations have been extensively used as a quote of the activity of transport systems [27]. Since the magnitude of the depolarizations reflects a net positive charge that crosses the membrane, in the case of anions, such as NO_3_^−^ or Pi, the membrane depolarization reveals the function of symport mechanisms fueled by a positive driving ion. Thus, to characterize the transport system mechanisms that mediate the high-affinity uptake of NO_3_^−^, amino acids and Pi in the seagrass *P. oceanica* the effect of micromolar concentration additions on membrane potential of mesophyll leaf cells was analyzed for each nutrient.

**Figure 1:**
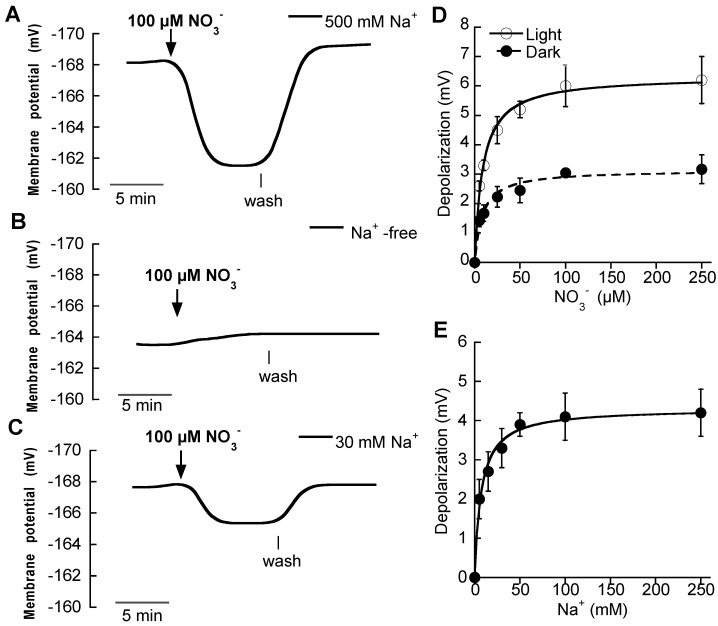
Effect of the addition of micromolar NO_3_^−^ concentrations on the membrane potential of *P. oceanica* mesophyll leaf cells. Membrane potential changes after of the addition of 100 μM NO_3_^−^ (downward arrow) in (**A**) ASW containing 500 mM NaCl, (**B**) in Na^+^ -free ASW and (**C**) in ASW containing 30 mM NaCl. Vertical lines indicate onset of NO_3_^−^ wash. (**D**) Mean values of membrane potential depolarizations induced by increasing NO_3_^−^ concentrations in ASW containing 500 mM Na^+^ under light (open symbols) or dark (closed symbols) conditions. (**E**) Mean values of membrane potential depolarizations induced by the addition of 100 μM NO_3_^−^ in ASW containing increasing Na^+^ concentrations. In D and E data were fitted to the Michaelis-Menten equation and kinetics parameters are shown in Table 2. Values are the means ± SD of five independent replicates.

Additions of micromolar concentrations of NO_3_^−^ evoked rapid plasma membrane depolarizations in mesophyll leaf cells of N-starved plants (Figure 1A). Measurable changes were evident even at 5 μM NO_3_^−^ and showed saturation at concentrations around 100 μM NO_3_^−^. As depolarizations are an integral function of the net charge carried by the NO_3_^−^ transport system, the concentration dependence of the depolarizations can be used for the estimation of the affinity of this system for NO_3_^−^. Fitting the data to the Michaelis-Menten equation yielded a *Km* value of 8.7 ± 1 μM NO_3_^−^ and a maximum depolarization of 6.3 ± 0.2 mV (Figure 1D, Table 2). On the other hand, the NO_3_^−^-induced depolarizations were lower in the dark than in the light, but also showed saturation kinetics with a similar *Km* value, 8.2 ± 1.7 μM NO_3_^−^ but with maximum depolarization reduced by half (Figure 1D, Table 2).

As we previously reported for *Z. marina* [4], NO_3_^−^-induced depolarizations were not detected in Na^+^-free ASW in *P. oceanica* mesophyll leaf cells. However, after the addition of 30 mM Na^+^ to the assay media, the NO_3_^−^-induced depolarization was recovered (Figure 1B,C). To investigate this Na^+^ -dependence, the depolarization induced by 100 μM NO_3_^−^ was assayed at different external Na^+^ concentrations. As expected for a Na^+^-dependent transport system, NO_3_^−^-restored depolarizations showed saturation kinetics at increasing Na^+^ concentrations (Figure 1E). Data were fitted to the Michaelis-Menten equation rendering a semisaturation constant of 7.2 ± 1.1 mM Na^+^ and a maximum restored depolarization of 4.3 ±0.1 mV, that was effectively saturated at concentrations around 50 mM Na^+^, supporting the Na^+^-dependence for the high-affinity NO_3_^−^ uptake in *P. oceanica* mesophyll leaf cells.

**Figure 2:**
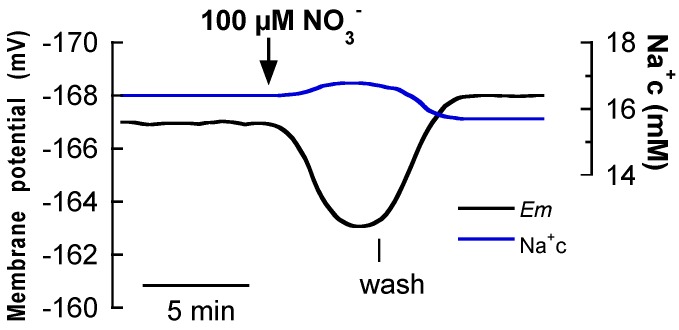
Effect of the addition of 100 μM NO_3_^−^ on membrane potential and cytosolic Na^+^ concentration of *P. oceanica* mesophyll leaf cells. Black trace shows the membrane potential (*Em*, mV) and the blue one corresponds to the cytosolic Na^+^ concentration (Na^+^c, mM) simultaneously measured. The downward arrow indicates the addition of NO_3_^−^ and the vertical line the onset of the wash. Traces are a representative example of three equivalent measurements.

To further analyze the NO_3_^−^/Na^+^ symport, the effect of NO_3_^−^ addition on the cytosolic Na^+^ concentration was measured. As shown Figure 2, the addition of 100 μM NO_3_^−^ simultaneously evoked a membrane depolarization and the increase of cytosolic Na^+^ (17 ± 2 mM Na^+^) by 0.4 ± 0.2 mM Na^+^. This effect is similar to that formerly found in the case of Na^+^-dependent high-affinity NO_3_^−^ transport in the seagrass *Z. marina* [5] revealing the coupling NO_3_^−^/Na^+^ uptake and suggesting the use of Na^+^ as driving ion for the high-affinity NO_3_^−^ uptake in those species. 

**Figure 3:**
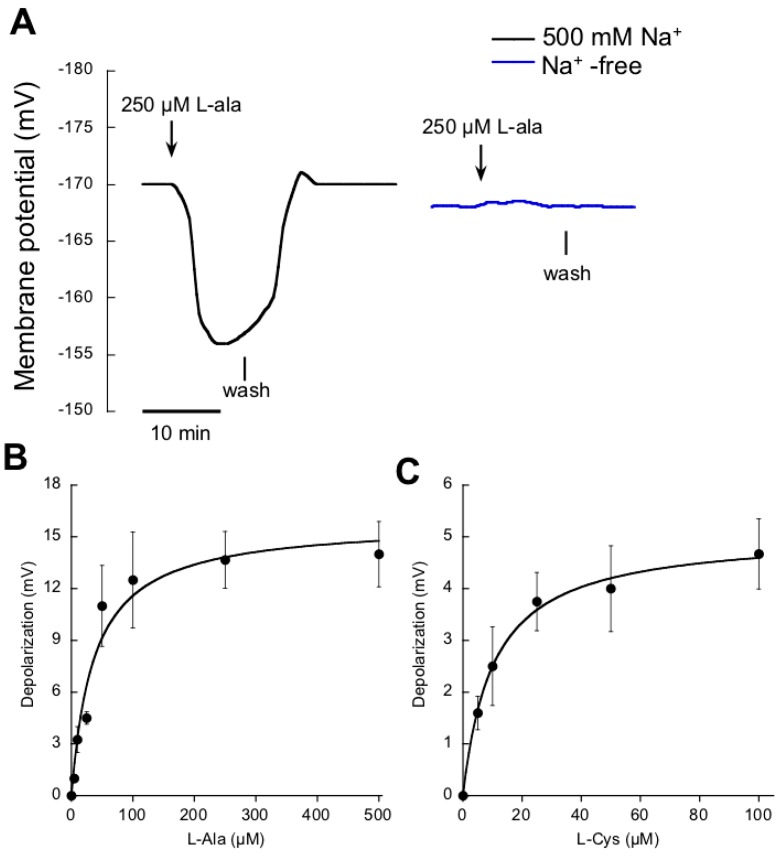
Effect of the addition of micromolar amino acid concentrations on the membrane potential of *P. oceanica* mesophyll leaf cells. (**A**) Membrane potential changes after the addition of 250 μM l-ala (downward arrows) in ASW containing 500 mM NaCl (black trace) or in Na^+^-free ASW (blue trace); vertical lines show the onset of the wash. Mean values of the membrane potential depolarizations induced by increasing concentrations of l-ala (**B**) or l-cys (**C**). Data were fitted to the Michaelis-Menten equation and kinetics parameters are shown in Table 2. Values are the means ± SD of five independent replicates.

To explore the existence of other transport systems fueled by Na^+^ in *P. oceanica*, the transport of the amino acids was also analyzed. After a survey among the complete list of them, the two that caused a significant response were alanine (l-ala) and cysteine (l-cys). As in the case of NO_3_^−^, the addition of micromolar concentrations of l-ala evoked rapid membrane depolarizations in mesophyll leaf cells of N-starved plants. These depolarizations were only recorded in ASW containing Na^+^ (Figure 3A). A similar response was observed in the case of micromolar additions of l-cys (data not shown). Since both amino acids exhibit equivalent pH value (6.1), at the pH of seawater (8.2) both solutes show a net negative charge; thus, to depolarize the membrane a net influx of positive charge is needed. As in the case of NO_3_^−^, Na^+^ -dependence of plasma membrane depolarizations strongly suggest that amino acid/Na^+^ symport systems operate at the plasma membrane of *P. oceanica*. In both cases, l-ala and l-cys, induced depolarizations showed saturation kinetics (Figure 3B,C) and values were fitted to the Michaelis-Menten equation, rendering a *Km* value of 37 ± 11 μM L-ala and 10±1 μM l-cys, respectively (Table 2). Cysteine transport exhibited lower saturation concentration (around 40 μM l-cys) and lower maximum depolarization than alanine kinetics (Table 2), which saturated at 100 μM l-ala.

### 2.3. Na^+^-Dependent High-Affinity Pi Uptake in Single Mesophyll Leaf Cells

**Figure 4:**
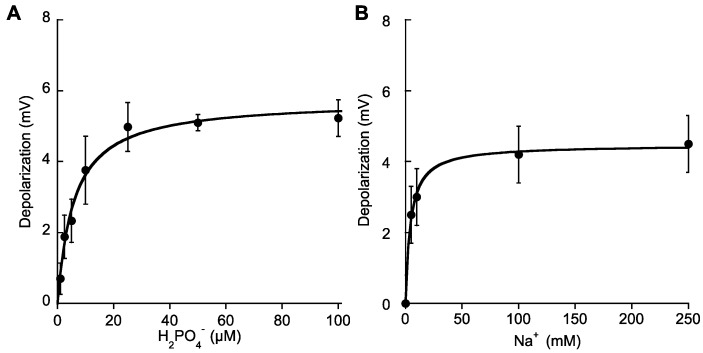
Membrane potential depolarizations induced by additions of micromolar H_2_PO_4_^−^ concentrations. (**A**) Mean values of membrane depolarizations induced by increasing Pi concentrations in ASW containing 500 mM Na^+^. (**B**) Mean values of the membrane potential depolarizations induced by the addition of 25 μM H_2_PO_4_^−^ in ASW containing increasing Na^+^ concentrations. Data were fitted to the Michaelis-Menten equation and kinetics parameters are shown in Table 2. Values are the means ± SD of five independent replicates.

In P-starved plants, additions of micromolar concentrations of Pi (5–100 μM H_2_PO_4_^−^) evoked rapid membrane depolarizations, unveiling the influx of a net positive charge. As found for other nutrients, no depolarizations were observed in Na^+^-free ASW (data not shown). Pi-induced depolarizations showed saturation kinetics and fitting of the data to the Michaelis-Menten equation rendered a *Km* value of 5.8 ± 1 μM H_2_PO_4_^−^ and a maximum depolarization of 5.7 ± 0.2 mV (Table 2). On the other hand, depolarizations induced by saturating phosphate concentrations (25 μM Pi) showed Na^+^ -dependence and rendered a saturation curve at increasing Na^+^ (Figure 4A). Semisaturation constant for Na^+^ was 4.3 ± 0.5 mM (Table 2), a lower value than the quoted for NO_3_^−^ transport, suggesting a higher efficiency for Pi transport than for NO_3_^−^ in *P. oceanica* mesophyll leaf cells. Maximum restored depolarization at increasing Na^+^ concentrations was 4.5 ± 0.1 mV, which effectively saturated at 50 mM Na^+^ (Figure 4B).

## 3. Discussion

*P. oceanica* leaves exhibit higher net rates of NO_3_^−^ and Pi uptake in the presence than in the absence of millimolar concentration of Na^+^. Among the seagrasses, this pattern has been only described in the case of *Zostera marina* [4,5]. In full strength Na^+^ artificial seawater, *P. oceanica* NO_3_^−^ and Pi net uptake rates show values in the same range than those quoted at saturating nutrient concentrations in different seagrasses, where, as observed in *P. oceanica*, slightly higher rates are observed for NO_3_^−^ (3.7–75 μmol NO_3_^−^ g^−1^ DW h^−1^) than for Pi uptake, 0.014–43 μmol g^−1^ DW h^−1^, [28]. The decrease of NO_3_^−^ and Pi uptake rates by *P. oceanica* leaves in the absence of Na^+^ strongly suggests the operation of Na^+^-dependent mechanisms for the uptake of both nutrients, as we have previously characterized in *Z. marina* [4,5]. 

Electrophysiological measurements show that NO_3_^−^ and Pi are incorporated by high-affinity transport systems in mesophyll leaf cells of *P. oceanica*. The semisaturation constant value, obtained from the uptake kinetics is lower for Pi than for NO_3_^−^, pointing out the higher affinity for Pi uptake in this species. In addition, the semisaturation constant for NO_3_^−^ transport is also higher than the reported value in single mesophyll leaf cells of *Z. marina* (*Km* = 2.3 ± 0.78 μM NO_3_^−^; [4]) but are much lower than those reported for the high-affinity NO_3_^−^ uptake in terrestrial plants, close to 60 μM NO_3_^−^ in barley [29] or Arabidopsis [30]; reviewed in [31]. Interestingly, apart from NO_3_^−^ uptake, *P. oceanica* mesophyll leaf cells also show a high-affinity transport of amino acids. Several evidences support that dissolved organic nitrogen utilization seems to be widespread among seagrasses, which provides these species with a competitive advantage over macroalgae in oligotrophic environments [32,33]. Saturation kinetics of amino acids had been previously reported for the tropical seagrasses *Thalassia hemprichii*, *Halodule uninervis*, and *Cymodocea rotundata*, showing *Km* values from 3.5 to 10.5 μM [34], which are similar to the value obtained in mesophyll leaf cells of *P. oceanica* for cysteine (10 ± 1 μM) but are lower than the *Km* for alanine (37 ± 11 μM). However, in contrast to the results of this work, no evidences for Na^+^-dependent amino acids uptake systems have been previously described in seagrasses.

In the case of terrestrial vascular plants, four transport systems have been shown to play a role in root acquisition of amino acids. These are amino the acid permeases AtAAP1 [35] and AtAAP5 [36,37], lysine/histidine-type transporter AtLHT1 [38,39], and the compatible solute/proline transporter AtProT2 [40]. LHT1 is probably the most important transporter for root uptake of l-Ala [37]. This high-affinity transporter is also localized at the plasma membrane of mesophyll cells and it has been demonstrated to be responsible for uptake of acidic and neutral amino acids from the leaf apoplast [38]. Interestingly, the *Km* values of the high-affinity amino acids transport found in *P. oceanica* leaf cells are similar to those reported in kinetics analysis of LHT1 transport activity, from 7.4 to 44.8 μM [37]. In terrestrial vascular plants this transporter functions as a general amino acid permease similar to other AAPs which, when expressed in yeast or oocytes, mediate H^+^-coupled, Na^+^-independent, uptake of a wide variety of amino acids, reviewed in [41].

Thus, Na^+^-dependent high-affinity alanine and cysteine transport mechanisms found in *P. oceanica* mesophyll leaf cells seem to support that secondarily adaptation of vascular plants to alkaline and high Na^+^ environments, such seawater, is based, together with other described adaptation processes, on exploiting the high inwardly directed electrochemical gradient for Na^+^, despite the widespread plasma membrane H^+^ economy in vascular plants. Na^+^-dependent high-affinity nutrient uptake mechanisms had been found in marine organisms as cyanobacteria [19,20], diatoms and several algae [21,22,23], but only in the case of our previous work in *Z. marina* it has been demonstrated to operate in vascular plants [4,5]. 

As observed in *Z. marina*, mesophyll leaf cells of *P. oceanica* also exhibit high-affinity Pi uptake. Semisaturation constant for Pi uptake kinetics is higher than that obtained in epidermal root cells of *Z. marina* (1.5 μM; [5]) but similar to the values reported in leaves of the seagrasses *Zostera noltii* (12.1 μM Pi; [42]), *Thalassia hemprichii* (7.7 μM Pi; [43]) or *Thalasia testudinum* (11.9 μM Pi; [44]) and in the range of the high-affinity *Km* values described among members of the H^+^-symporter (PHS) family in vascular terrestrial plants [45]. However, as it has been discussed in the case of NO_3_^−^ and amino acids, results shown in this work demonstrated that a Na^+^-coupled high-affinity Pi transport system operates at the plasma membrane of mesophyll leaf cell of *P. oceanica*. Thus, the appearance of Na^+^-dependent high-affinity transporters could be a general adaptation model for nutrient uptake in seagrasses and could be considered as a strategy to inhabit salinized, alkaline and P and N-poorly media such as seawater.

In the case of terrestrial vascular plants, it has been a long-standing hypothesis that the plasma membrane H^+^-ATPase provides the energy for membrane transport and therefore for nutrient uptake [46]. Plasma membrane H^+^-ATPase is an electrogenic pump that exports protons from the cytosol and, in addition, generates a transmembrane electrochemical gradient of H^+^, and all plasma membrane secondary transporters exploit the energy accumulated in the membrane to drive ion movements [47]. Nevertheless this model seems to be different in seagrasses. *P. oceanica* leaf cells, as was previously reported for *Z. marina*, exhibit a quite constant and low cytosolic Na^+^ concentration (16 ± 1 mM; [25] and this work). This value is in the range of those measured in terrestrial glycophytes, but much lower than the proposed values for terrestrial halophytes, between 70 and 200 mM Na^+^ in plants grown in the presence of 50 and 400 mM Na^+^, respectively [48]. Despite the absence of Na^+^-ATPases, which are not found in vascular plants [6], the maintenance of the low cytosolic Na^+^ concentrations found in seagrasses seems to be the resultant of both, the very low plasma membrane Na^+^/K^+^ permeability ratio (0.003 in *Z. marina*, [17]; 0.005 in *P. oceanica*, our data, unpublished) and the operation of an active Na^+^ efflux mechanism at the plasma membrane [24]. In fact, considering the high Na^+^ concentration (500 mM) of the seawater and the rather negative membrane potential (−174 mV; [25]) measured in *P. oceanica* leaf cells, the inwardly directed Na^+^ electrochemical gradient (in millivolts) results -260 mV, two-fold higher than the H^+^ motive force value (−122 mV) calculated in *P. oceanica*, considering the cytosolic pH of mesophyll leaf cells (7.3, [25]) and the seawater pH (8.2).

**Figure 5:**
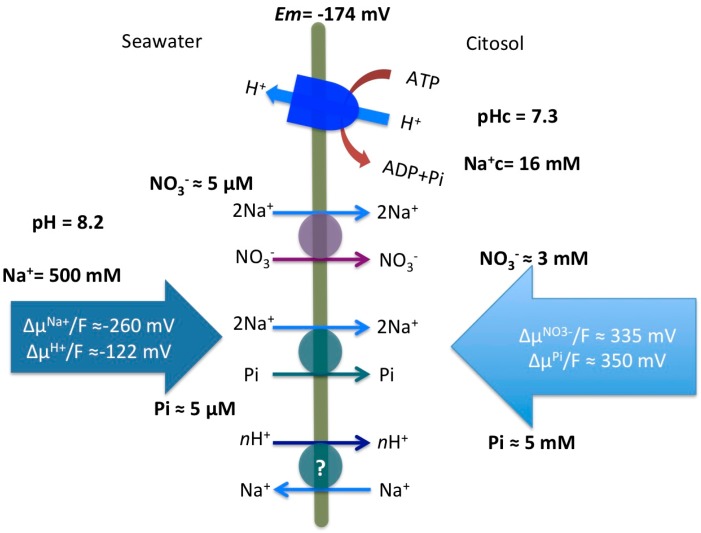
Energization model and stoichiometry for plasma membrane high-affinity NO_3_^−^ and Pi transporters in mesophyll leaf cells of *P. oceanica.* A Na^+^/H^+^ antiporter is also indicated as a putative mechanism to maintain the sodium gradient. Cytosolic and seawater ion concentrations and the calculations of the electrochemical potential gradients, included on the big arrows (∆μ/F; mV), are discussed in the text.

As shown in Figure 5, comparisons of Na^+^ electrochemical gradient against those calculated for NO_3_^−^ and Pi provide an estimation of Na^+^ stoichiometry of the transport systems. Considering the low NO_3_^−^ and Pi concentrations of the Mediterranean Sea and cytosolic concentrations of NO_3_^−^ (3 mM) and Pi (5 mM) similar to the values previously discussed for *Z. marina* [5], around 340 mV are needed to energize the transport of NO_3_^−^ and Pi into *P. oceanica* leaf cells. This means that a stoichiometry of 2Na^+^/NO_3_^−^ and 2Na^+^/Pi would render enough energy to make the transport thermodynamically feasible (Figure 5). Na^+^-dependence of both high-affinity transport systems is half-saturated at low millimolar Na^+^ concentrations (7.2 ± 1.1 mM and 4.3 ± 0.5 mM, for NO_3_^−^ and Pi uptake, respectively). These values are higher than the *Km* for Na^+^ reported in *Z. marina* for the high-affinity NO_3_^−^ transport (0.72 mM Na^+^; [4]) and are also higher than the value reported for Na^+^/NO_3_^−^ transport in the marine diatom *Phaeodactylum tricornutum* (2.58 mM Na^+^; [22]) or the Na^+^ semisaturation constant for Na^+^ of the high-affinity HCO_3_^−^ transporters found in cyanobacteria (1 and 1.7 mM; [19]). In any case, these low-millimolar *Km* values for Na^+^ imply that the NO_3_^−^ and Pi high-affinity transporters of *P. oceanica*, as well as those of *Z. marina*, would be functioning at saturating Na^+^ concentrations in seawater.

## 4. Materials and Methods 

### 4.1. Plant Material and Assay Solutions

Plants of *Posidonia oceanica* (L.) Delile plants were collected, at 2 m depth, in Punta de Calaburras, Málaga, southern Spain (36°30′23.4′′ N 4°38′37.6′′ W). In each sampling, several leaves (10–15) attached to a piece of the rhizome were transported to the laboratory in a thermos container in less than 30 min. Leaves were then placed in an aquarium filled with continuously aerated natural seawater. Temperature was held at 15 °C. Plants were illuminated at a light intensity of 150 μmol photons m^−2^ s^−1^ with a photoperiod 16 L/8 D. Seawater was renewed every 3 days and leaves were used for experiments within 2 weeks after sampling. 

Plants were nitrogen starved for at least 15 days prior to experiments on NO_3_^−^ and amino acids transport in N-free artificial seawater (ASW) containing 0.01 mM NaH_2_PO_4_. On the other hand, plants were starved of phosphate for at least 5 d in P-free ASW containing 0.01 mM NaNO_3_ before assays on Pi transport were carried out. The composition of ASW was: 500 mM NaCl, 55 mM MgCl_2_, 12 mM CaCl_2_, 10 mM KCl, 10 mM NaHCO_3_, and pH was adjusted to pH 8.2 with 1 N NaOH.

For depletion experiments and membrane potential measurements ASW was buffered with 10 mM MOPS-Bis tris Propane, pH 8.2. In Na^+^-free ASW, NaCl was replaced by 800 mM Sorbitol or 500 mM Cl-choline, keeping a similar osmolality (1.09 osmol Kg^−1^; cryoscopic osmometer, Osmomat, model 030, Gonotec GmbH, Berlin, Germany) and HCO_3_^−^ was added as HCO_3_K. NO_3_^−^ and H_2_PO_4_^−^ were added as sodium salts, or as potassium salts when using Na^+^-free media. All chemicals were purchased from Sigma-Aldrich, Darmstadt, Germany.

### 4.2. Depletion Experiments

Excised leaves (0.3–0.6 g fresh weight) were placed separately in 250 mL flasks and incubated in 50 mL ASW containing 500 mM NaCl or in the absence of Na^+^ (Na^+^-free ASW). The assay was carried out at 25 °C with gentle and constant agitation. At the beginning of the experiment, 100 μM NO_3_K or 10 μM KH_2_PO_4_ were added to the assay medium and samples were taken at 0, 5, 10, 15, 30 min, 1, 2, 4, 8, 12 and 24 h. NO_3_^−^ or Pi concentrations were analyzed colorimetrically in each sample [49,50]. Net uptake rates were estimated as the slope of the linear phase of NO_3_^−^ or Pi depletion curves. Three replicates were conducted for each assay.

### 4.3. Electrophysiology

Membrane potential (*Em*) was measured using the standard glass microelectrode technique described by Felle [51]. As previously reported for *Zostera marina* [4,5,17,24,52] or *Posidonia oceanica* [25] leaf pieces (1.5 cm length), in which the epidermis had been partially removed, were mounted in plexiglass chambers (volume 1.1 mL) and connected to a continuous gravity-based flow through system of the assay medium, maintained at a constant flux rate (10 mL min^−1^) during the measurements.

For membrane potential measurement, mesophyll leaf cells were impaled with single-barreled microelectrodes. Microelectrodes were backfilled with 500 mM KCl and fixed to electrode holders, containing an Ag/AgCl pellet, that were connected to a high- impedance differential amplifier (FD-223a, World Precision Instruments, Sarasota, FL, USA).

For simultaneous measurements of membrane potential and cytosolic sodium, Na^+^-selective microelectrodes based on double-barreled capillaries were used. Details of pulling and backfilling can be found in our previous works [5,24,25]. In summary, ionophore ETH227 (Fluka 71176), dissolved in PVC/THF (4% *w*/*v*) was used as Na^+^ sensor. The backfilling solution was 0.5 M NaCl and the difference signal was calibrated against 1–500 mM NaCl solutions, prepared in 96 mM KCl to minimize K^+^ interference. Calibration slopes were close to 45 mV pNa^+^. 

### 4.4. Data Presentation and Analysis

Time-course measurements are shown as single traces, representative of a number of equivalent experiments carried out under the same conditions, as stated in the figure legends. Data are given as mean ± SD, and the number of repetitions (*n*) is indicated in every experiment. Membrane depolarization data were fitted to the Michaelis-Menten equation using a non-linear regression computer program (KaleidaGraph, Synergy Software, Reading, PA, USA). Data were analyzed using SPSS Statistics (Armonk, NY, USA), version 21. The significance level was set at *p* < 0.05. 

## 5. Conclusions

Classical depletion experiments, membrane potential and cytosolic Na^+^ measurements demonstrated that high-affinity NO_3_^−^, amino acid and Pi uptake are mediated by Na^+^-dependent transport mechanisms operating in the plasma membrane of mesophyll leaf cells of *P. oceanica*. Thus, the existence of Na^+^-coupled high-affinity uptake systems could be a general model for the mineral nutrition of seagrasses and could be considered a strategy to inhabit salinized, alkaline and low-nutrient environment. However, further investigation is needed to identify the molecular identity of these transporters, transport mechanisms and their occurrence in other seagrass species. 

## Figures and Tables

**Table 1:** NO_3_^−^ and Pi net uptake rates in leaves of *P. oceanica*. N- or P-starved plants were incubated in artificial seawater (ASW) containing 500 mM NaCl and Na^+^ -free ASW in which NaCl was substituted by 0.8 M Sorbitol, in the case of NO_3_^−^ depletion assays, or 0.5 M Cl-choline in the case of Pi depletion. Net uptake rates were calculated as the slope of the linear phase of NO_3_^−^ and Pi depletion curves within the first 24 h after the addition of 100 μM NO_3_K and 10 μM PO_4_H_2_K, respectively. Data are mean ± SD of three independents replicates.

Net Uptake Rate	ASW	Na^+^-Free ASW
μmol NO_3_^−^·g_FW_^−1^·h^−1^	0.34 ± 0.01	0.09 ± 0.01 *
μmol Pi·g_FW_^−1^·h^−1^	0.04 ± 0.003	0.01 ± 0.002 *

* Asterisks indicate significant differences at *p* < 0.05 (Student’s *t*-test).

**Table 2:** NO_3_^−^, amino acids and Pi kinetics parameters in mesophyll leaf cells of *P. oceanica*.

Nutrient	*Km* (μM)	*Dmax* (mV)
NO_3_^−^ (Light)	8.7 ± 1 (7.2 ± 1.1 mM Na^+^) ^1^	6.3 ± 0.2
NO_3_^−^ (Dark)	8.2 ± 1.7	3.1 ± 0.1
l-ala	37 ± 11	16 ± 1.2
l-cys	10 ± 1	5 ± 0.2
Pi	5.8 ± 1 (4.3 ± 0.5 mM Na^+^) ^1^	5.7 ± 0.2

^1^ Values in brackets show semisaturation constant for Na^+^ as transport driving ion. Data are mean ± SD of five independents experiments.

## Abbreviations

ASW	Artificial Sea Water
*Dmax*	Maximum Depolarization
*Em*	Membrane Potential
